# Extracellular micronutrient levels and pro-/antioxidant status in trauma patients with wound healing disorders: results of a cross-sectional study

**DOI:** 10.1186/1475-2891-12-157

**Published:** 2013-12-05

**Authors:** Sandra C Blass, Hans Goost, Christof Burger, René H Tolba, Birgit Stoffel-Wagner, Peter Stehle, Sabine Ellinger

**Affiliations:** 1 Department of Nutrition and Food Sciences, Nutritional Physiology, University of Bonn, Endenicher Allee 11-13, 53115 Bonn, Germany; 2 Department of Orthopedics and Trauma Surgery, University Hospital of Bonn, Sigmund-Freud-Str. 25, 53127 Bonn, Germany; 3 Institute of Laboratory Animal Science, University Hospital Aachen, Pauwelstr. 30, 52074 Aachen, Germany; 4 Department of Clinical Chemistry and Clinical Pharmacology, University Hospital Bonn, Sigmund-Freud-Str. 25, 53127 Bonn, Germany; 5 Faculty of Food, Nutrition and Hospitality Sciences, Hochschule Niederrhein, University of Applied Sciences, Rheydter Str. 277, 41065 Mönchengladbach, Germany

**Keywords:** Disorders in wound healing, Trauma patients, Nutritional status, Micronutrients, Vitamins, Oxidative stress, Inflammation

## Abstract

**Background:**

Disorders in wound healing (DWH) are common in trauma patients, the reasons being not completely understood. Inadequate nutritional status may favor DWH, partly by means of oxidative stress. Reliable data, however, are lacking. This study should investigate the status of extracellular micronutrients in patients with DWH within routine setting.

**Methods:**

Within a cross-sectional study, the plasma/serum status of several micronutrients (retinol, ascorbic acid, 25-hydroxycholecalciferol, α-tocopherol, β-carotene, selenium, and zinc) were determined in 44 trauma patients with DWH in addition to selected proteins (albumin, prealbumin, and C-reactive protein; CRP) and markers of pro-/antioxidant balance (antioxidant capacity, peroxides, and malondialdehyde). Values were compared to reference values to calculate the prevalence for biochemical deficiency. Correlations between CRP, albumin and prealbumin, and selected micronutrients were analyzed by Pearson’s test. Statistical significance was set at *P* < 0.05.

**Results:**

Mean concentrations of ascorbic acid (23.1 ± 15.9 μmol/L), 25-hydroxycholecalciferol (46.2±30.6 nmol/L), β-carotene (0.6 ± 0.4 μmol/L), selenium (0.79±0.19 μmol/L), and prealbumin (24.8 ± 8.2 mg/dL) were relatively low. Most patients showed levels of ascorbic acid (<28 μmol/L; 64%), 25-hydroxycholecalciferol (<50 μmol/L; 59%), selenium (≤ 94 μmol/L; 71%) and β-carotene (<0.9 μmol/L; 86%) below the reference range. Albumin and prealbumin were in the lower normal range and CRP was mostly above the reference range. Plasma antioxidant capacity was decreased, whereas peroxides and malondialdehyde were increased compared to normal values. Inverse correlations were found between CRP and albumin (*P* < 0.05) and between CRP and prealbumin (*P* < 0.01). Retinol (*P* < 0.001), ascorbic acid (*P* < 0.01), zinc (*P* < 0.001), and selenium (*P* < 0.001) were negatively correlated with CRP.

**Conclusions:**

Trauma patients with DWH frequently suffer from protein malnutrition and reduced plasma concentrations of several micronutrients probably due to inflammation, increased requirement, and oxidative burden. Thus, adequate nutritional measures are strongly recommended to trauma patients.

## Background

Disorders in wound healing (DWH) are frequently observed post-surgically in patients with vascular diseases and soft tissue trauma [1]. DWH are associated with a prolonged hospital stay and essentially contribute to high morbidity and mortality [2]. Thus, apart from the individual burden, DWH generate enormous costs in the health care system.

The pathophysiological mechanisms leading to DWH are not completely understood. Recent studies in patients with pressure ulcers [3] support the hypothesis that general protein/energy malnutrition can considerably increase the risk for DWH by several mechanisms. The lack of energy and nitrogen containing metabolites, such as amino acids, may hamper wound healing (WH) by diminishing the body’s capacity for cell repair. Low food/energy intake is often related to an insufficient provision with essential micronutrients. It is well-known that several vitamins and trace elements, such as retinol, ascorbic acid, 25-hydroxycholecalciferol (25(OH)D_3_), and zinc, are involved in collagen synthesis, cell division and epithelialization [4-7]. Thus, nutrient intake below the actual recommendations published for healthy subjects may increase the risk to develop DWH or even aggravate already existing DWH. Moreover, it is still under debate whether the metabolic needs for micronutrients are considerably higher in trauma compared with physiological conditions [3]. An insufficient intake of micronutrients may lead to intra-/extracellular deficiencies resulting in an imbalance between pro-/and antioxidants which exerts cytotoxic effects and, consequently, may impair WH as shown in a small patient group for selenium [8]. However, representative cross-sectional studies focusing on the assessment and evaluation of general and specific nutritional status in a variety of patients with DWH are scarce.

Therefore, the aim of this cross-sectional study was to assess general nutritional status, micronutrient profile and the concentration of selected biomarkers of pro-/antioxidative balance in trauma patients with DWH in a routine clinical setting.

## Methods

### Patients

Following a mono-center cross-sectional design, adult trauma patients with DWH (defined as failure to heal, i.e. wound not closed or persisting secretion within ten days after trauma or surgery) were consecutively recruited between May and December 2006 at the Department of Orthopedics and Trauma Surgery, University of Bonn. Exclusion criteria were defined as follows: parenteral and enteral nutrition, exclusive implant removal, pressure ulcers as primary diagnosis, HIV infection, chronic inflammatory bowel diseases, liver diseases, drug abuse, known pregnancy, lactation, stay in the intensive care unit and sepsis. After enrolment, data on main diagnosis, comorbidities and medication were obtained and the individual injury severity score [9] was determined. The time between trauma/surgery and enrolment was documented. All patients received hospital food provided by an external caterer. Patients were asked whether they supplemented vitamins, zinc and/or selenium.

All patients provided written, informed consent prior to enrolment. The study was conducted according to the Declaration of Helsinki, 2004, and was authorized by the Ethics Committee of Bonn University (No. 029/06).

### Blood sampling

On the day after enrolment, blood samples were collected after an overnight fast in EDTA or lithium coated tubes and in tubes without anticoagulant. Plasma and serum were obtained within two hours by centrifugation at 2000 × g, 4°C for 10 min. EDTA plasma for analysis of ascorbic acid and heparinized plasma for analysis of malondialdehyde (MDA) were prepared as described earlier [10,11]. All samples were stored at -80°C until analysis. Laboratory parameters, except for those investigated routinely, were analyzed in duplicate.

### Clinical chemistry

Leukocytes (flow cytometry, Sysmex, Norderstedt, Germany), C-reactive protein (CRP; nephelometry, Siemens Healthcare Diagnostics, Eschborn, Germany), and cholesterol (polychromatic measurement, Siemens Healthcare Diagnostics) were analyzed by routine clinical chemistry. The reference value for CRP was obtained from the Department of Clinical Chemistry and Clinical Pharmacology, University Hospital Bonn.

### Anthropometric data and nutritional status

Body mass index (BMI, kg/m^2^) was determined by weighing the patients and asking for their height as the height could not be determined in each patient. The BMI was evaluated according to the WHO criteria (underweight: <18.5; normal weight: 18.5 – 24.9; overweight: 25.0 – 29.9; obesity: ≥ 30.0) [12]. Calf and upper arm circumferences (cm) were measured in duplicate. Reference values for calf and upper arm circumferences (sex- and age-dependent) were taken from the NHANES study [13]; patients with values below the 10^th^ percentile were categorized as malnourished (Table 1). General nutritional status and disease-associated weight loss were determined by Subjective Global Assessment (SGA) [14]. Patients were classified as well-nourished (SGA A), moderately malnourished or suspected to be malnourished (SGA B) or as severely malnourished (SGA C) (Table 2). In all cases, anthropometric data and general nutrition status were determined by the same investigator (SCB).

**Table 1:** Anthropometric data

	**Patients**^**a**^	**Patients below RR**	**Patients above RR**	**Reference range**
Body mass index (kg/m^2^)	25.5 ± 5.3	11%	52%	18.5 – 24.9
Calf circumference (cm)	34.8 ± 5.1	24%	0%	> 10th P.
Upper arm circumference (cm)	30.5 ± 4.9	19%	0%	> 10th P.

^a^Data are means ± SDs, based on 42 patients for body mass index and on 37 patients for calf and upper arm circumference. P.: percentile; RR: reference range.

**Table 2:** Nutritional status determined by the subjective global assessment

	**Absolute prevalence *(n)***	**Relative prevalence (%)**
SGA A	20	45
SGA B	15	34
SGA C	9	21

Data are based on 44 patients. Categories: *SGA* A, well nourished; SGA B, moderately malnourished or suspected of being malnourished; SGA C, severely malnourished. General malnutrition is reflected by SGA categories B/C.

Plasma concentrations of retinol (CV 4.3%), ascorbic acid (CV 3.2%), α-tocopherol (CV 4.1%), and β-carotene (CV 3.2%) were measured in EDTA-plasma by HPLC [15,16]. 25(OH)D_3_ was analyzed by ELISA (CV 5.6% according to manufacturer; IDS, Frankfurt/Main, Germany). Vitamin E status was determined as α-tocopherol to cholesterol ratio. Zinc was measured in heparinized plasma by photometry (CV 1.9%; Wako Chemicals, Neuss, Germany) and selenium by atom absorption spectrometry (CV 3%; Biosyn, Fellbach, Germany). Albumin was analyzed in serum by routine clinical chemistry (Department of Clinical Chemistry and Clinical Pharmacology, University Hospital Bonn). Prealbumin was determined in heparinized plasma by radial immunodiffusion (CV 0.7%; The Binding Site, Schwetzingen, Germany). Reference ranges published recently for retinol [17], ascorbic acid [17], α-tocopherol [18], β-carotene [17], albumin [19], and prealbumin [20] are included in Table 3. The reference ranges for zinc and selenium (Table 3) were obtained from the Department of Clinical Chemistry and Clinical Pharmacology, University Hospital Bonn.

**Table 3:** Micronutrient status in plasma and plasma protein concentrations

	**Patients**	**Reference range**
Micronutrients		
Retinol (μmol/L)	1.4 ± 0.7	0.7 – 1.75
Ascorbic acid (μmol/L)	23.1 ± 15.9	28 – 85
25-Hydroxycholecalciferol (nmol/L)	46.2 ± 30.6	50 – 175
α-Toc/chol ratio (μmol/mmol)	9.8 ± 3.2	> 2.2
β-Carotene (μmol/L)	0.6 ± 0.4	0.9 – 4.6
Zinc (μmol/L)	12.4 ± 3.2	11.5 – 19.4
Selenium (μmol/L)	0.79 ± 0.19	> 0.94
Proteins		
Albumin (g/dL)	32.8 ± 8.5	Age-related^a^
Prealbumin (mg/dL)	24.8 ± 8.2	15 – 36

Data are means ± SDs, based on the data of 44 subjects for all parameters except for zinc (*n* = 37), selenium (*n* = 42), and albumin (*n* = 39).

^a^Reference range for albumin [g/L]: 35 – 53 (≤ 60 y), 34 – 48 (> 60 y), 33 – 47 (> 70 y), 31 – 45 (> 80 y). α-Toc/chol ratio: α-Tocopherol-to-cholesterol ratio.

### Markers of pro-/antioxidant balance

The trolox equivalent antioxidant capacity (TEAC) assay [21] was used to determine antioxidant capacity (CV 1.2%) in EDTA-plasma. MDA was measured in plasma by photometry (CV 7.4%) [22]. An ELISA kit was used to determine the concentration of peroxides (Dr. Franz Tatzber KEG, Bisamberg, Austria; CV 4.3% according to the manufacturer) in EDTA-plasma. Uric acid was analyzed in serum by photometry (Siemens Healthcare Diagnostics). The plasma of several groups of staff members was analyzed to obtain values for TEAC (*n* = 89), peroxides (*n* = 21), and MDA (*n* = 33) from healthy controls as reference values of these parameters are still lacking. For uric acid, the reference range was obtained from the Department of Clinical Chemistry and Clinical Pharmacology, University Hospital Bonn.

### Evaluation strategy and statistics

Data on TEAC, peroxides, and MDA were checked for normality by Kolmogorov-Smirnov test. If normality was not reached, even after log-transformation, comparison between patients and healthy subjects was done by Mann–Whitney *U* test. Correlations between CRP, albumin and prealbumin and selected micronutrients were analyzed by Pearson’s test. Statistical significance was assumed for *P* < 0.05. PASW software, version 17.0 (SPSS Inc., Munich, Germany), was used for statistical evaluation. Results are presented as means and standard deviations and as median and quartiles, respectively.

## Results

### Patients

Forty-four trauma patients with DWH based on the predefined clinical criteria were included in this study. For demographic and clinical data, see Table 4. Most patients (68%) had a soft tissue trauma and/or infections. About one third suffered from vascular diseases or diabetes mellitus. Twelve patients were smokers. Only a minority (*n* = 4) used nutritional supplements by own decision. Infections of osteosynthesis material occurred in five and infections with methicillin-resistant *Staphylococcus aureus* in three patients. Two patients suffered from osteomyelitis. Median period between trauma and study entry was 26 days (interquartile range: 15 – 68 days).

**Table 4:** Demographic and clinical data

	**Patients (*n*** **= 44)**
Sex (male/female) (*n*)	29/15
Age (y)^a^	60 ± 21
Age (<65 y/≥65 y) (*n*)	22/22
Period between trauma and study entry (d)^b^	26 (15; 68)
Main diagnosis (*n*)	
Long bone fracture	10
Joint fracture	14
Pelvic fracture	6
Miscellaneous fracture	14
Injury severity score^a^	14 ± 8
Soft tissue trauma (*n*); (grade: I°/II°/III°)	11 (5/4/2)
Infection (*n*)	30
Comorbidities (*n*)	
Vascular diseases	13
Diabetes mellitus	7
Atopic dermatitis	3
Cortisone intake (*n*)	7
Current intake of nutritional supplements *(n)*^c^	4
Smoker (*n*)	12
Length of hospital stay (d)^b^	29 (21; 67)

^a^means ± SDs, ^b^median (quartiles); ^c^vitamins, minerals, and combinations, ingested by own decision.

### Clinical chemistry

Mean leukocyte count was 8.1 ± 2.2 G/L and median CRP concentration 21.2 [6.4; 88.7] mg/L. Only four patients had CRP concentrations within the reference range (0 to <3 mg/L).

### Body composition and nutritional status

As shown in Table 1, mean BMI was 25.5 ± 5.3 kg/m^2^. Eleven percent of the patients were underweight, 37% were normal weight, 31% were overweight and 21% were obese. Twenty-four percent of the patients had a calf circumference below the 10th percentile, while an upper arm circumference below the 10th percentile was identified in 19% of the patients. General malnutrition (SGA B/C) was prevalent in 55% of the patients. As shown in Table 2, 15 patients (34%) were at risk for malnutrition or suspected to be malnourished (SGA B) and nine patients (21%) were judged as severely malnourished (SGA C). Significant weight loss (> 10% of weight within six months) was observed in 18% of the patients with DWH.

**Figure 1:**
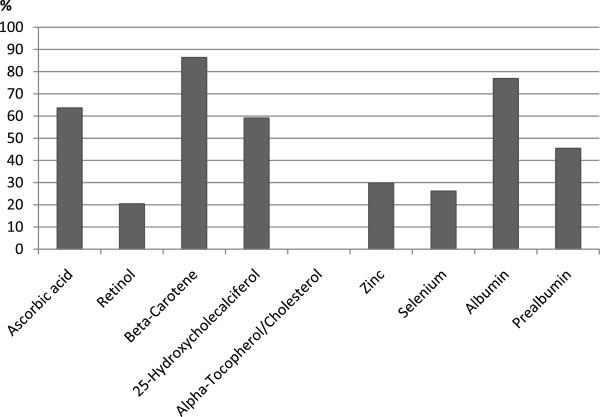
**Prevalence of micronutrients and plasma protein concentrations below the reference range.** Data were based on 44 patients except for zinc (*n* = 37), selenium (*n* = 42), and albumin (*n* = 39). Reference ranges: ascorbic acid 28 – 85 μmol/L, retinol 0.7 – 1.75 μmol/L, β-carotene 0.9 – 4.6 μmol/L, 25-Hydroxycholesterol > 50 - 175 nmol/l, α-tocopherol/cholesterol > 2.2 μmol/mmol, zinc 11.5 - 19.4 μmol/L, selenium > 0.94 μmol/L, albumin [g/L] 35 – 53 (≤ 60 y), 34 – 48 (> 60 y), 33 – 47 (> 70 y), 31 – 45 (> 80 y), and prealbumin >15 mg/dL. None of the patients had an α-tocopherol status below the reference range.

Results on plasma protein and micronutrient concentrations are shown in Table 3. The concentrations of ascorbic acid, 25(OH)D_3_, β-carotene, and selenium were relatively low, and in most patients below the reference range (Figure 1). A deficiency was also observed for albumin and, partly, for prealbumin. The prevalence of low plasma values of pro-/vitamins, selenium, zinc as well as albumin and prealbumin did not differ between generally well-nourished (SGA A) and malnourished (SGA B/C) patients (data not shown).

As expected, there was an inverse correlation between CRP and albumin (r = -0.358, *P* = 0.025) and between CRP and prealbumin (r = -0.426, *P* = 0.004). Retinol (r = -0.526, *P* < 0.001), ascorbic acid (r = -0.406, *P* < 0.007), zinc (r = -0.654, *P* < 0.001), and selenium (r = -0.585, *P* < 0.001) were negatively correlated with CRP.

### Markers of pro-/antioxidant balance

Results on TEAC, peroxides and MDA are shown in Table 5. In patients with DWH, TEAC was lower (*P* < 0.001), and the concentrations of peroxides (*P* < 0.006) and MDA (*P* < 0.001) were higher compared to healthy controls. Uric acid concentration (280±85 μmol/L) was within the reference range (154 - 357 μmol/L).

**Table 5:** Markers of pro-/antioxidant balance and of oxidative stress

	**Patients (*n*** **= 44)**	**Healthy controls**^**a**^	***P*-value**^**b**^
TEAC (mmol TE/L)	1.24 ± 0.16	1.60 ± 0.16	< 0.001
Peroxides (mmol/L)	0.90 ± 0.27	0.72 ± 0.10	0.006
Malondialdehyde (μmol/L)	83.7 ± 33.5	14.3 ± 4.1	< 0.001

Data are means ± SDs. ^a^Determined in healthy controls from our staff (*n* = 89 for TEAC, *n* = 21 for peroxides, and *n* = 33 for malondialdehyde). ^b^for group comparison (Mann–Whitney *U* test). TEAC: trolox equivalent antioxidant capacity; TE: trolox equivalents.

## Discussion

To the best of our knowledge, this is the first cross-sectional study collecting various anthropometric, biochemical and clinical data to evaluate nutritional status and body composition in a large variety of post-surgical patients with DWH. The mono-center study was performed within clinical routine ensuring that all patients received comparable medical therapy, care and dietetic measures. Thus, the evaluation of selected clinical and biochemical markers may provide indication for the failure of WH.

Despite the fact that most patients were normal weight, overweight or obese and had calf and upper arm circumferences within the reference range (Table 1), general malnutrition according to SGA was prevalent in most patients (Table 2). Parameters reflecting long-term (albumin) and short-term (prealbumin) protein supply (Table 3, Figure 1) were in the lower normal range. Hence, general malnutrition which has been proposed to favor DWH [3] may have contributed to the development of DWH.

Mean plasma concentrations of ascorbic acid, 25(OH)D_3_, β-carotene and selenium (Table 3) were below the reference values and 59 - 86% of the patients had a deficiency in these micronutrients (Figure 1). Since post-traumatic and post-surgical metabolic events, such as inflammation [23,24] and oxidative stress [25], may generally contribute to lower plasma levels of ascorbic acid and β-carotene, low plasma status of these micronutrients in trauma patients with DWH may be a concomitant phenomenon considering increased levels of CRP, MDA, and peroxides. While it may not be the reason for the development of DWH, it nevertheless should be considered that the plasma level of micronutrients does not necessarily reflect the micronutrient status in the wounded tissue with respect to substrate fluxes to the wound area. Such fluxes have been observed in rats for free amino acids during early wound healing period, leading to a relative lack of arginine in whole body [26]. Thirty percent of the patients exhibited an insufficient serum zinc status (Figure 1). Comparably low concentrations of zinc transporters, such as albumin and prealbumin, (Table 3) may at least partly explain this observation. Another line of reasoning is the acute phase response (APR) itself [27,28]. Elevated concentrations of acute phase proteins, such as CRP and interleukin-6, are known to increase the expression of the zinc importer Zip14 [29] which leads to a fast zinc redistribution to organs [30]. Zorilla et al. showed that the serum zinc level is predictive for WH [5,31]. They determined the serum zinc level in preoperative patients before elective hip replacement which may explain the different results obtained in our study (post-surgical analysis). In contrast to other micronutrients, such as retinol, ascorbic acid, and zinc, selenium does not have a direct physiological function in WH. However, as a cofactor of glutathione peroxidase, selenium may reduce oxidative stress in patients with DWH [11]. Thus, the high prevalence of selenium concentrations below the reference range in patients with DWH (Figure 1) may contribute to DWH [11]. Insufficient serum 25(OH)D_3_ concentrations were observed in most of our patients (Figure 1). Since Miller et al. [32] observed an inverse association between serum vitamin D levels and inflammatory response following hip fracture, low 25(OH)D_3_ concentrations in our patients may result from APR-mediated inflammation. Moreover, immobilization of the patients may contribute to the low vitamin D status due to the lack of UV-induced endogenous synthesis.

Disturbances in the redox state are discussed to be risk factors for delayed WH [8]. Since direct measurements of the short-lived reactive oxygen species require laborious and expensive techniques, such as electron paramagnetic resonance, indirect methods are used in routine clinical setting to detect an imbalance between pro- and antioxidants [33]. This includes analysis of peroxidation products, such as MDA and peroxides, analysis of single antioxidants (e.g., ascorbic acid, α-tocopherol, β-carotene) [34] and analysis of total antioxidant capacity reflecting the synergistic action between endogenous (albumin, uric acid) and nutritive (ascorbic acid, α-tocopherol and β-carotene) antioxidants [35]. Oxidative stress in patients with DWH was indicated by increased concentrations of peroxides compared to healthy adults. TEAC was lower in patients with DWH than in healthy controls (Table 5), probably due to lower concentrations of several endogenous (e.g., albumin) and exogenous antioxidants like ascorbic acid (Table 3) with a high contribution to plasma antioxidant capacity. Despite the relatively low specificity of MDA, [36], the increased values of MDA suggest an increased lipid peroxidation in patients with DWH, which is in line with increased peroxides and reduced TEAC.

The strength of this study is the broad variety of biochemical, anthropometric, and clinical parameters which provides a detailed picture on the micronutrient status of trauma patients with DWH. Unfortunately, data on the quantity and quality of the diet considering energy, protein, and micronutrients of interest and data on the kind and dose of supplemented micronutrients were not collected. Hence, the impact of the nutritional intake on the nutritional status of our patients cannot be assessed. The most serious limitation of our study refers to the study design as a cross-sectional study which does not allow any conclusion whether DWH originated from nutritional deficiency or not. A further limitation refers to the high prevalence of vascular diseases, diabetes mellitus and wound infections (Table 4), which are known risk factors for DWH [1]. Hence, effects on WH from these classic risk factors cannot be ruled out. Due to these limitations, it is difficult to judge the role of selected micronutrients for WH *in vivo*. In future studies with a prospective design, participants should be comparable with regard to classic risk factors for DWH (e.g., diabetes mellitus). Also, more attention should be paid to time-dependent changes following trauma and/or surgery.

## Conclusions

Trauma patients with DWH frequently suffer from protein malnutrition and a biochemical deficiency in several micronutrients, probably due to inflammation, increased requirement and oxidative burden. Thus, tailored nutritional measures (fresh fruit, vegetables and high quality protein) and/or early supplementation with selected micronutrients are strongly recommended to hospitalized trauma patients. 

## Abbreviations

APR: Acute phase respone; BMI: Body mass index; CRP: C-reactive protein; DWH: Disorders in wound healing; 25(OH)D3: 25-hydroxycholecalciferol; MDA: Malondialdehyde; SGA: Subjective global assessment; TEAC: Trolox equivalent antioxidant capacity; WH: Wound healing.

## Competing interests

The authors declare that they have no competing interests.

## Authors’ contributions

SCB, SE, PS, and HG contributed to the conception and the design of the study. SCB was responsible for data acquisition and performed statistical analysis. HG recruited the patients. RHT was responsible for the analysis of malondialdehyde and BSW for the analysis of biomarkers determined within clinical chemistry. CB was the clinical advisor. SCB, SE, PS, and HG interpreted the data and drafted the manuscript. All authors read and approved the final manuscript.
